# Cold hypersensitivity in the hands and feet is associated with erectile dysfunction in young Taiwanese men

**DOI:** 10.1038/s41598-024-60260-x

**Published:** 2024-05-08

**Authors:** Che-Jui Chang, Yu-Hua Fan, Yi-Chun Chiu, Wei-Ming Cheng

**Affiliations:** 1 Division of Urology, Department of Surgery, Zhongxiao Branch, Taipei City Hospital, Taipei, Taiwan; 2 Department of Urology, College of Medicine, National Yang Ming Chiao Tung University, Taipei, Taiwan; 3 Shu-Tien Urological Research Center, National Yang Ming Chiao Tung University, Taipei, Taiwan; 4 Department of Urology, Taipei Veterans General Hospital, Taipei, Taiwan; 5 Department of Exercise and Health Sciences, University of Taipei, Taipei, Taiwan

**Keywords:** Cold hypersensitivity in hands and feet, Erectile dysfunction, Young men, Taiwanese men, Endothelial dysfunction, Autonomic dysregulation, Sexual dysfunction, Quality of life, Risk factors

## Abstract

Cold hypersensitivity in the hands and feet (CHHF) is a protective or predisposing factor for many diseases; however, the relationship between CHHF and erectile dysfunction (ED) remains unclear. We aimed to investigate associations between CHHF and ED among young men of Southeast Asian origin. In this cross-sectional study, sexually active Taiwanese men aged 20–40 years were enrolled via an online questionnaire comprising general demographic information, comorbidities, subjective thermal sensations of their hands and feet in the past 6 months, and their erectile function using the International Index of Erectile Function-5 (IIEF-5). Participants who reported cold sensation of hands and feet were classified to have CHHF; those with IIEF-5 score ≤ 21 were considered to have ED. Total 54.2% and 27.9% of participants had ED and CHHF, respectively. Men with CHHF were significantly younger, had lower body mass index and IIEF-5 scores (*p* < 0.001), and a lower prevalence of diabetes mellitus (*p* = 0.033) along with higher prevalence of ED, psychiatric disorders, and insomnia (*p* < 0.001). After adjusting for predisposing factors of ED, CHHF (odds ratio 1.410, 95% confidence interval 1.159–1.714; *p* = 0.001) remained an independent predictor of ED. Thus, CHHF is independently associated with ED, affecting more than a quarter of young Taiwanese men. Autonomic dysregulation and subclinical endothelial dysfunction may be common pathophysiologies of CHHF and ED.

## Introduction

Erectile dysfunction (ED) is defined as “the inability to achieve or maintain an adequate erection for satisfactory sexual performance”^1^. Although the prevalence of ED increases with age, complaints of this condition among younger men are becoming increasingly frequent, which may have a negative effect on their quality of life. ED can be classified as organic (neurogenic, hormonal, arterial, cavernosal, and drug-induced), psychogenic, or mixed type (psychogenic and organic)^2^. ED was considered to be psychogenic in nature in most men under 40 until recently, but studies have shown that 15–72% of them have organic etiologies^3^, especially vasculogenic ED caused by anomalies in penile arterial inflow or venous outflow^4^.

Cold hypersensitivity in the hands and feet (CHHF) is a physical condition in which there is a sensation of noxious cold in an individual’s extremities even under conditions that would not typically evoke such a sensation. CHHF is the most commonly observed in people of East Asian ethnicity, especially women, and has a significant impact on health-related quality of life^5,6^. Although the precise mechanism of CHHF remains unknown, it has been linked to a heritable phenotype^5^ and hypersensitive vasoconstrictor response of the terminal vessels^7^. CHHF has been reported to be either a protective or a predisposing factor for many diseases; however, the relationship between CHHF and ED remains unclear.

Theoretically, hypersensitive vasoconstriction of the terminal vessels of the internal iliac artery, namely, the penile artery, negatively affects erectile function. CHHF, caused by contraction of blood vessels in the extremities due to psychological stress, neurovascular disease, or medical factors^8^, can increase the risk of developing ED^2,9^. Conversely, CHHF has also been reported to be associated with lower rates of metabolic and cardiovascular diseases including hypertension (HTN), diabetes mellitus (DM), impaired fasting glucose, dyslipidemia, stroke, fatty liver, and angina pectoris^10^, which are known risk factors of ED^2,9^. As the potential effects of CHHF on ED are contradictory, there is a need to clarify the relationship between these two pathologies. This study examined the relationship between ED and CHHF in a large cohort of young Taiwanese men. We believe that the findings of the present study will expand our understanding of the pathophysiology of ED, especially in young men.

## Methods

### Study population and design

This online cross-sectional study was conducted for 1 month from December 1 to December 31, 2022 (The average temperature was 17.9 degrees Celsius according to the Central Weather Administration in Taiwan) in accordance with the principles of the Declaration of Helsinki and was approved by the Ethics Committee of Taipei City Hospital (IRB number: TCHIRB-11107012-E). Written informed consent for participation was not required for this study in accordance with the national legislation and the institutional requirements.

A questionnaire was developed and distributed using the SurveyCake software (25sprout, Taipei, Taiwan). Adult Taiwanese men (20–40 years) who could read and write Mandarin characters were invited to participate in the study through a link on social media handles such as Facebook, Instagram, X (formerly Twitter), and YouTube. Those without sexual contact with at least one person in the previous 6 months and undergoing phosphodiesterase 5 inhibitors (PDE5i) treatment for ED were excluded. To prevent the same person from posting multiple responses with the same login and password, Internet protocol address filtering was utilized. Additionally, form refilling was avoided by including a verification question at the beginning of the questionnaire.

### Questionnaire

The questionnaire comprised questions concerning demographic data such as age, body mass index (BMI), smoking history, and regular exercise habits. A BMI ≥ 27.5 kg/m^2^ was defined as obesity according to the World Health Organization expert consultation^11^. History of smoking was defined as smoking cigarettes or e-cigarettes currently or in the past^12^. We inquired about the participants’ style, frequency, and intensity of exercise. Participants were classified as having regular exercise habits if they engaged in regular resistance training twice a week or 40 min of aerobic exercise at a moderate to vigorous intensity four times per week for more than 3 months^13^. Additionally, we also asked the participants to self-report their comorbidities diagnosed in the hospital, including HTN, DM, hyperlipidemia, psychiatric disorders (PD, including major depressive and anxiety disorders), and insomnia. Medication history was obtained, and the participants undergoing PDE5i treatment for ED were excluded. The International Index of Erectile Function-5 (IIEF-5) scores were used for ED assessment; there are five questions in the IIEF-5, with each question being scored in a 1–5 Likert-type scale, with higher and lower scores indicating better and worse erectile functions, respectively. Thus, the total score ranges between 5 and 25, and participants with IIEF-5 scores ≤ 21 were considered to have ED^14^.

We also asked the participants to rate the thermal sensations in their hands and feet in the past 6 months as cold, intermediate, or warm. Those who responded "cold" to both hands and feet were defined to have CHHF, while those who answered "warm" or "intermediate" to both hands or feet were classed as the non-CHHF group. Those who answered "I don't know" or "cold" to only one of these two questions were excluded from the present study^10,15^.

### Statistical analyses

Categorical parameters were expressed as numbers (percentages), while continuous parameters were expressed as mean ± standard deviation. Pearson’s chi-square test and Student's *t*-test were used to compare the differences in categorical and continuous parameters between the CHHF and non-CHHF groups, respectively. Univariate and multivariate logistic regression analyses were used to explore the predictive factors for ED. A variable was included in the multivariate model if it had a *p* value < 0.05 in the univariate analysis, or if it was reported as a risk factor for ED in previous studies^2^. All statistical analyses were performed using the SPSS Statistics version 22 for Windows (IBM Corp., Armonk, NY, USA). Statistical significance was set at *p* < 0.05.

### A Ethical approval

The online cross-sectional study was approved by the Ethics Committee of Taipei City Hospital (IRB number: TCHIRB-11107012-E) prior to the initiation of the study in December 2022.

## Results

Of the 2,199 Taiwanese men aged 20–40 years included in the study with a questionnaire response rate of 36.3%, 613 (27.9%) were classified into the CHHF group. The demographic characteristics of the participants are presented in Table 1. The age and BMI of men in the CHHF group were significantly lower than that of men in the non-CHHF group (age and BMI: *p* < 0.001). Total comorbidities between the two groups showed no significant differences (*p* = 0.053); however, the incidence of DM was significantly higher in the non-CHHF group (*p* = 0.033). The PD and insomnia rates were significantly higher in the CHHF group than in the non-CHHF group (*p* < 0.001). No differences in the prevalence of HTN or hyperlipidemia were observed between the two groups. Notably, prevalence of current or past smoking was significantly higher in the CHHF group than in the non-CHHF group (*p* = 0.016). IIEF-5 scores were significantly lower in the CHHF group (*p* < 0.001), and participants in the CHHF group were significantly more likely to have ED (*p* < 0.001). Although the prevalence of mild ED was higher in the non-CHHF group, mild-to-moderate, moderate, and severe ED were more prevalent in the CHHF group.

**Table 1:** Demographic data: Non-CHHF group and CHHF group (20–40 years old).

Parameters	Total	Non-CHHF	CHHF	*p*-value
Participants (*n*, %)	2199 (100)	1586 (72.1)	613 (27.9)	–
Age (years, Mean ± SD)	31.8 ± 5.2	32.1 ± 5.1	31.0 ± 5.3	< 0.001*
BMI (kg/m^2^, Mean ± SD)	24.8 ± 4.3	25.4 ± 4.4	23.0 ± 3.4	< 0.001*
Comorbidities (*n*, %)	396 (18.0)	270 (17.0)	126 (20.6)	0.053
Hypertension	110 (5.0)	82 (5.2)	28 (4.6)	0.561
Diabetes mellitus	44 (2.0)	38 (2.4)	6 (1.0)	0.033*
Hyperlipidemia	84 (3.8)	66 (4.2)	18 (2.9)	0.179
PD & insomnia	243 (11.1)	147 (9.3)	96 (15.7)	< 0.001*
Smoking (n, %)	430 (19.6)	290 (18.3)	140 (22.8)	0.016*
Regular exercise (*n*, %)	1198 (54.5)	872 (55.0)	326 (53.2)	0.447
Resistance training	725 (33.0)	521 (32.8)	204 (33.3)	0.848
Aerobic exercise	850 (38.7)	623 (39.3)	227 (37.0)	0.331
IIEF	20.1 ± 4.3	20.4 ± 4.0	19.2 ± 4.8	< 0.001*
Erectile dysfunction (*n*, %)	1191 (54.2)	826 (52.1)	365 (59.5)	< 0.001*
Mild	772 (35.1)	564 (35.6)	208 (33.9)	
Mild-to-moderate	303 (13.8)	203 (12.8)	100 (16.3)	
Moderate	88 (4.0)	48 (3.0)	40 (6.5)	
Severe	28 (1.3)	11 (0.7)	17 (2.8)	

Data are shown as mean ± SD or numbers (percentages). CHHF, cold hypersensitivity in the hands and feet; SD = standard deviation; BMI = body mass index; PD = psychiatric disorder; IIEF** = **International Index of Erectile Function. *Significant values are in bold.

In the univariate analysis (Table 2), age ≥ 30 years, PD, insomnia, lack of regular exercising habits (especially no regular aerobic exercise), and CHHF were significantly correlated with ED. After adjusting for age, obesity, smoking history, comorbidities, and exercise habits, age (odds ratio [OR] 1.327, 95% confidence interval [CI] 1.106–1.592; *p* = 0.002), obesity (OR 1.271, 95% CI 1.021–1.583; *p* = 0.032), PD and insomnia (OR 1.594, 95% CI 1.202–2.115; *p* = 0.001), lack of regular exercise habits (OR 1.192 95% CI 1.003–1.417; *p* = 0.046), and CHHF (OR 1.410, 95% CI 1.159–1.714; *p* = 0.001) remained independent predictors of ED among young Taiwanese men.

**Table 2:** Logistic regression analyses of variables associated with erectile dysfunction (IIEF ≤ 21) among participants aged 20–40 years.

Variable	Univariate logistic regression analysis		Multivariate logistic regression analysis	
	Crude OR (95% CI)	*p*-value	Crude OR (95% CI)	*p*-value
Age (years)				
< 30	1	ref		
≥ 30	1.285 (1.076–1.285)	0.006*	1.327 (1.106–1.592)	0.002*
BMI (kg/m^2^)				
< 27.5	1	ref		
≥ 27.5	1.228 (1.000–1.509)	0.05	1.271 (1.021–1.583)	0.032*
Comorbidities				
No	1	ref		
Yes	1.344 (1.077–1.676)	0.009*		
Hypertension				
No	1	ref		
Yes	1.188 (0.805–1.751)	0.386	0.992 (0.653–1.507)	0.970
Diabetes mellitus				
No	1	ref		
Yes	1.116 (0.611–2.039)	0.721	0.942 (0.493–1.799)	0.856
Hyperlipidemia				
No	1	ref		
Yes	1.078 (0.695–1.673)	0.737	0.939 (0.587–1.501)	0.791
PD & insomnia				
No	1	ref		
Yes	1.628 (1.233–2.149)	0.001*	1.594 (1.202–2.115)	0.001*
Smoking				
No	1	ref		
Yes	1.061 (0.859–1.312)	0.582	0.976 (0.786–1.211)	0.825
Regular exercise				
Yes	1	ref		
No	1.211 (1.023–1.433)	0.026*	1.192 (1.003–1.417)	0.046*
Resistance training				
Yes	1	ref		
No	1.048 (0.877–1.253)	0.606		
Aerobic exercise				
Yes	1	ref		
No	1.226 (1.032–1.456)	0.021*		
CHHF				
No	1	ref		
Yes	1.354 (1.121–1.636)	0.002*	1.410 (1.159–1.714)	0.001*

IIEF** = **International Index of Erectile Function; OR = odds ratio; CI = confidence interval; BMI = body mass index; PD = psychiatric disorder; CHHF, cold hypersensitivity in the hands and feet. *Significant values are in bold.

## Discussion

This study aimed to investigate the relationship between CHHF and ED in young Taiwanese men. CHHF occurs in about 20–52% of the Eastern Asian population, particularly in women^16,17^. The prevalence of CHHF in men has been reported to be approximately 10.4–44.3%^5,6,10,15,18^, which is comparable to the 27.9% incidence found in our study. In the present study, CHHF was found to be associated with increased prevalence of ED (59.5%) among young Taiwanese men. Age ≥ 30 years, obesity (BMI ≥ 27.5 kg/m^2^), PD and insomnia, lack of regular exercise habits, and CHHF were independent predictors of ED in young men. Among all participants in the present study, 54.2% were found to have ED, which was substantially higher than that reported previously^19^. To our knowledge, this is the first study to explore the association between CHHF and ED.

In the present study, the mean age and BMI were lower in the CHHF group, which is consistent with earlier studies^5,10,15,18^. CHHF is known to be associated with a lower waist circumference and waist-to-hip ratio in men^18^. One study examined the temperatures of the third fingernail bed of the right hand in obese (BMI ≥ 30 kg/m^2^) and normal-weight (BMI = 18–25 kg/m^2^) adults using infrared thermography. The mean fingernail bed temperature was significantly higher in the obese participants than in the normal-weight participants. The increased layer of fat insulation in obese men could reduce core-to-skin heat loss, which could prevent them from developing CHHF^21^.

Participants in the CHHF group of our study had significantly higher incidence of smoking history; however, no significant difference was found among Korean men for smoking in a study by Bae et al.^6^. We defined smokers as those who smoked conventional cigarettes or e-cigarettes currently or in the past, as in other studies; however, Bae et al.^6^ did not clarify their definition of current smokers. The effects of smoking duration and e-cigarette smoking on CHHF remain unknown. Moreover, we only recruited young men (mean age: 31.8 years), while the male participants in Bae et al.’s study were older (mean age: about 45 years). All these factors may have resulted in the inconsistent relationship between smoking and CHHF in our study and Bae et al*.*’s study. Cigarette smoke contains nicotine, carbon monoxide, oxidants, and metals that can damage the endothelium, and thus impair endothelial vasodilation. Increased sympathetic activation due to cigarette smoke may also be a potential contributing factor for CHHF^12^.

In our study, the patients with CHHF had a significantly lower incidence of DM. This was similar to the findings of another study by Bae et al.^10^. Cold stress has been reported to induce adiponectin secretion in the white adipose tissue, leading to diet-induced thermogenesis through elevated glucose utilization, thereby reducing the prevalence of hyperglycemia among men with CHHF^18^.

We also found no differences in the incidence of HTN and dyslipidemia between the CHHF and non-CHHF participants, similar to the findings of Park and Cha^18^. However, other studies have indicated that people with CHHF have a lower prevalence of HTN, dyslipidemia, and risk of metabolic syndrome^10,18^. This may be because those studies predominantly involved women, whereas, we recruited only young men. We also found that the participants with CHHF had significantly higher rates of PD and insomnia than those without CHHF. We speculated that patients with PD would have a higher sympathetic tone^22^, resulting in vasoconstriction of the terminal vessels of the hands and feet. Those with CHHF would also have difficulty falling asleep because of the uncomfortable coldness of their hands and feet when lying in bed. It has been reported that the degree of peripheral blood vessel dilatation of the hands and feet is a good physiological predictor of rapid onset of sleep^23^. Another study showed that the distal–proximal skin temperature gradient is closely associated with a prolonged onset of sleep^24^.

Individuals with CHHF experience higher psychological stress^8^, which results in variable functional disorders^10,15^ that are also associated with ED^9^. By contrast, individuals with CHHF have a lower incidence of HTN, DM, dyslipidemia, stroke, fatty liver, and angina pectoris^10^, which reduces the risk of ED^2,9^. The conflicting association between ED and CHHF piqued our interest in this study. We found that CHHF was an independent predictor of ED after adjusting for age, BMI, comorbidities, smoking status, and regular exercise habits. Although participants with CHHF have a reduced prevalence of DM, the harmful impact of psychological stress related to CHHF can overwhelm the beneficial impact of CHHF on glucose metabolism, resulting in the development of ED in the young population.

CHHF can be regarded as dormant Raynaud's phenomenon (RP) without changes in the color of the terminal extremities^5^. RP is associated with reduced skin blood flow, which is exacerbated by cold temperatures or emotional stress. Increased sympathetic receptor activation in blood vessels, endothelial dysfunction, increased concentration of endothelin-1 (ET-1), and various anomalies in the central thermoregulatory system have been hypothesized to be potential contributors to the development of primary RP^25^. RP is associated with a high mortality rate due to cardiovascular disease. Similar to ED, RP may be a marker of an undiagnosed vascular disease^26^. Symptoms of RP are similar to those of primary vascular dysregulation (PVD). Vascular dysregulation refers to the regulation of blood flow which is not adapted to the needs of the respective tissues. Vasospasm can cause a reduction in body temperature anywhere on the surface of the body, not only on the hands, feet, or nose, but also at the scrotum. The sensation of cold extremities, a leading symptom of PVD, is supported by finger skin temperature measurements. Participants with PVD tend to have cold extremities, signs of oxidative stress, and slightly elevated ET-1 plasma levels. A likely basis for PVD is endothelial dysfunction, which results in an imbalance in endothelium-derived vasoregulatory factors with high ET-1 and low nitric oxide (NO) plasma levels. In addition to vascular endotheliopathy, the autonomic nervous system is also compromised. This affects young men in particular, and testosterone has been proposed to play a role^27^. An examination of heart rate variability revealed sympathetic predominance in healthy PVD participants^28^. A study comparing slow- and normal-handed rewarmers showed that slow rewarmers have lower parasympathetic nervous system activity than normal rewarmers during the cold provocation test^29^. The pathophysiology of ED is similar to that of PVD. Although erectile function in youth is usually viewed as psychogenic, organic ED, especially arteriogenic ED, is common^3,19^. Arteriogenic ED is caused by autonomic dysregulation, endothelial dysfunction, and changes in smooth muscle function. Endothelial NO generation is considered the most important factor for the immediate relaxation of penile vessels and the corpus cavernosum, which results in the erection of the penis^2^. We hypothesized that high ET-1 and low NO plasma levels, which signify autonomic dysregulation and endothelial dysfunction, would be a common pathophysiology of CHHF and ED in young men.

The present study has some limitations. First, because connections between ED and CHHF were investigated using a cross-sectional methodology, no causative relationship could be identified. Therefore, a longitudinal investigation is warranted to ascertain pertinent association directions. Second, the study focused on sexually active men with ED (aged 20–40 years), which may have limited its applicability to older men with more risk factors for ED. Third, the study sample was recruited from social media rather than community settings, which may have introduced a selection bias^20^. Compared with the average online survey response rate of 44.1%^30^, the response rate was relatively low in the present study. The length of the survey, poor visual presentation, invitation designs, pre-notification and reminders, uninvited responses or multiple responses, and erratic internet coverage negatively impacted on the response rate^31^. The questionnaire was distributed through a link on social media, resulting in a higher browsing rate, thus lowering the response rate. However, with a sample size of at least 500, Fosnacht et al.^32^ discovered that the data remained reliable even with a 5%–10% response rate. Internet users with ED tend to look up information associated with ED and are thus more likely to complete the online questionnaire from the information acquired in the present study, resulting in a higher prevalence of ED (54.2%) in the present study. Nevertheless, since 98–99% of Taiwanese adults aged 20–40 years use the Internet^33^, recruiting young participants for related studies through social media platforms may be a suitable and economical approach. Fourth, we used questions to acquire subjective temperature sensations in the extremities, similar to other studies^5,6,10,15,18^. We did not objectively measure the temperature of the participants’ extremities, which is not feasible in an online survey. However, the definition of CHHF is simply subjective sensations rather than objective temperatures of the extremities. Despite these limitations, this study presented some interesting outcomes. In clinical practice, the risk factors for ED are difficult to identify in most young patients. Autonomic dysregulation and subclinical endothelial dysfunction may cause ED and CHHF in young patients. CHHF may be a sign of ED and vascular disease. Young men with CHHF should be vigilant about cardiovascular health and erectile function. Early life style modification and health education for young men with CHHF may be important. Moreover, we excluded participants receiving PDE5i in this study, so the beneficial impact of PDE5i on men with CHHF remained unknown. In addition, herbal medicines used in the management of CHHF may have the potential to be used to treat ED in young patients^34^. Our study contributes to the existing literature, setting the foundation for further studies that can clarify the causative relationship between ED and CHHF and the treatment of CHHF in ED.

## Conclusions

Our study indicates that CHHF is a common condition among young Taiwanese men, accounting for 27.9% of study participants. After adjusting for age, obesity, smoking history, comorbidities, and exercise habits, CHHF remained a significant predictor of ED among the youth. Autonomic dysregulation and subclinical endothelial dysfunction may be common pathophysiologies of CHHF and ED. Further studies are warranted to clarify the causative relationship between ED and CHHF.

## Data Availability

The datasets generated and analyzed during the current study are available from the corresponding author on reasonable request. Questionnaire of CHHF and ED: https://reurl.cc/b91pNd.

## References

[1] NIH Consensus Conference. Impotence. NIH Consensus Development Panel on Impotence. *Jama* **270**, 83–90 (1993). 8510302

[2] Shamloul R, Ghanem H (2013). Erectile dysfunction. Lancet.

[3] Ludwig W, Phillips M (2014). Organic causes of erectile dysfunction in men under 40. Urol. Int..

[4] Yafi FA (2016). Erectile dysfunction. Nat. Rev. Dis. Primers.

[5] Hur YM (2012). Feeling of cold hands and feet is a highly heritable phenotype. Twin. Res. Hum. Genet..

[6] Bae KH, Lee Y, Go HY, Kim SJ, Lee SW (2019). The relationship between cold hypersensitivity in the hands and feet and health-related quality of life in Koreans: A nationwide population survey. Evid. Based Complement Alternat. Med..

[7] Nagashima K (2002). Thermal regulation and comfort during a mild-cold exposure in young Japanese women complaining of unusual coldness. J. Appl. Physiol..

[8] Bae KH (2018). The definition and diagnosis of cold hypersensitivity in the hands and feet: Finding from the experts survey. Integr. Med. Res..

[9] Romano L (2022). Sexual dysfunction in patients with chronic gastrointestinal and liver diseases: A neglected issue. Sex Med. Rev..

[10] Bae KH (2018). The association between cold hypersensitivity in the hands and feet and chronic disease: results of a multicentre study. BMC Complement Altern. Med..

[11] Tan KCB (2004). Appropriate body-mass index for Asian populations and its implications for policy and intervention strategies. Lancet.

[12] Allen MS, Tostes RC (2023). Cigarette smoking and erectile dysfunction: an updated review with a focus on pathophysiology, e-cigarettes, and smoking cessation. Sex. Med. Rev..

[13] Gerbild H, Larsen CM, Graugaard C, Areskoug Josefsson K (2018). Physical activity to improve erectile function: A systematic review of intervention studies. Sex Med..

[14] Rosen RC, Cappelleri JC, Smith MD, Lipsky J, Peña BM (1999). Development and evaluation of an abridged, 5-item version of the International Index of Erectile Function (IIEF-5) as a diagnostic tool for erectile dysfunction. Int. J. Impot. Res..

[15] Bae KH (2016). Cold Hypersensitivity in the hands and feet may be associated with functional dyspepsia: Results of a multicenter survey study. Evid. Based Complement Alternat. Med..

[16] Lee S, Lee K, Song B (1996). The literature review on the cold hypersensitivity of women. Korean J. Obstet. Gynecol..

[17] Ushiroyama T, Kajimoto Y, Sakuma K, Ueki M (2005). Assessment of chilly sensation in Japanese women with laser doppler fluxmetry and acceleration plethysmogram with respect to peripheral circulation. Bull. Osaka Med. Coll..

[18] Park AY, Cha S (2017). Effects of cold sensitivity in the extremities on circulating adiponectin levels and metabolic syndrome in women. BMC Complement Altern. Med..

[19] Nguyen HMT, Gabrielson AT, Hellstrom WJG (2017). Erectile dysfunction in young men-a review of the prevalence and risk factors. Sex Med. Rev..

[20] Eysenbach G, Wyatt J (2002). Using the Internet for surveys and health research. J. Med. Internet Res..

[21] Savastano DM (2009). Adiposity and human regional body temperature. Am. J. Clin. Nutr..

[22] Yamamoto K, Shinba T, Yoshii M (2014). Psychiatric symptoms of noradrenergic dysfunction: A pathophysiological view. Psychiatry Clin. Neurosci..

[23] Kräuchi K, Cajochen C, Werth E, Wirz-Justice A (1999). Warm feet promote the rapid onset of sleep. Nature.

[24] Vollenweider S, Wirz-Justice A, Flammer J, Orgül S, Kräuchi K (2008). Chronobiological characterization of women with primary vasospastic syndrome: body heat loss capacity in relation to sleep initiation and phase of entrainment. Am. J. Physiol. Regul. Integr. Comp. Physiol..

[25] Cooke JP, Marshall JM (2005). Mechanisms of Raynaud's disease. Vasc. Med..

[26] Nietert PJ (2015). Raynaud phenomenon and mortality: 20+ years of follow-up of the Charleston Heart Study cohort. Clin. Epidemiol..

[27] Flammer J, Konieczka K, Flammer AJ (2013). The primary vascular dysregulation syndrome: implications for eye diseases. Epma J..

[28] Flammer J (2013). The eye and the heart. Eur. Heart J..

[29] Brändström H (2013). Autonomic nerve system responses for normal and slow rewarmers after hand cold provocation: effects of long-term cold climate training. Int. Arch. Occup. Environ. Health.

[30] Wu M-J, Zhao K, Fils-Aime F (2022). Response rates of online surveys in published research: A meta-analysis. Comput. Human Behav. Rep..

[31] Fan W, Yan Z (2010). Factors affecting response rates of the web survey: A systematic review. Comput. Human Behav..

[32] Fosnacht K, Sarraf S, Howe E, Peck LK (2017). How important are high response rates for college surveys?. Rev. Higher Educ..

[33] Cheng W, Fan YH, Liou YJ, Hsu YT (2021). The predictive factors of nocturia in young Asian adult males: An online survey. Sci. Rep..

[34] Yu JS, Lee D, Hyun D, Chang SJ (2018). Herbal medicines for cold hypersensitivity in the hands and feet: A systematic review and meta-analysis. J. Altern. Complement Med..

